# Nanotechnology in Therapeutics: hydrogels and beyond

**DOI:** 10.1186/1477-3155-5-5

**Published:** 2007-07-26

**Authors:** OV Salata

**Affiliations:** 1 The Wellcome Trust Centre for Human Genetics, Roosevelt Drive, Oxford, OX3 7BN, UK

## Abstract

Nanotechnology in Therapeutics: Current Technology and Applications, Edited by Nicholas A. Peppas, J. Zach Hilt and J. Brock Thomas (Horizon Bioscience, 2007) contains seventeen chapters written by leading specialists in the field of polymeric materials for drug delivery and holds wealth of background as well as state of the art material divided into four sections: "Intelligent Therapeutics and Responsive Delivery Systems for Improved Absorption and Delivery", "Therapeutic Micro- and Nanodevices", "Nanostructured Therapeutic Materials" and "Nanoparticulate Systems in Intelligent Therapy". This newly published volume provides a stimulating read and a good point of reference to researchers wishing to explore the interdisciplinary fusion of nnanotechnology and medical therapeutics. The following gives brief summary and critically reviews the book.

## Background

### Intelligent therapeutics and responsive delivery systems for improved absorption and delivery

The very first chapter of this section overviews the "concepts of medical chronobiology, chronopharmacology and chronotherapeutics" as the basis for applications of drug delivery technology" and is dealing with the drug delivery matching to the biological rhythms. Can a doctor who takes decisions on the drug dosage by monitoring patient's progress be replaced by an automated system (see Chapter 2, dedicated to the "Feedback Control in Drug delivery")? Four diseases are analysed in this context and only in the simplest single input case of glucose control in diabetes a feedback control seems to be feasible at present. One of the major non-technical hurdles in this field is the acceptance of the technology by both clinicians and patients.

The advances in drug delivery for nanoparticles are discussed in the third chapter of this thrilling volume. The main reason for creating nanoparticles is to improve solubility and bioavailability of drugs. It is a well known phenomenon that nanoparticles can be taken much faster across the cell membranes. There are also opportunities here to create a targeted drug delivery. Routes of nanoparticle preparation are described, followed by the discussion of the requirements to their physical and chemical properties for effective delivery.

Molecular recognition is one of the central concepts in biology and is of great importance for the creation of active synthetic nanomaterials. In the Chapter 4 ("Synthetic Ligand-Receptor Interactions in Delivery Systems") the authors are describing what is involved in the design of a synthetic receptor (as opposed to designing a ligand, which is a more common pharmaceutical problem). The next chapter is entitled "Nanoscale Analysis of Mucus-Carrier Interactions for Improved Drug Absorption". It describes the structure of the mucous layer on the molecular level and discusses the interaction of this layer with synthetic polymers.

The final sixth chapter of the "intelligent therapeutics" part is dedicated to the polymeric gene delivery vectors. The use of viral vectors for gene therapy is efficient but there are obvious safety concerns. The polymeric carriers are not very efficient at present but can have low cytotoxicity and immunogenicity. The future gene-polymeric complexes should be multifunctional and targeted to a specific disease.

### Therapeutic micro- and nanodevices

This section contains three chapters. The first one is entitled "Biohybrid Materials for Therapeutic Devices". Smart biomaterials incorporating sensing and actuating moieties would allow the therapeutic devices to response to the changes in the metabolism of an individual. Currently changes in pH are sensed and result in a mechanical action (drug release). Hydrogels are a very promising class of materials for this application. Some exciting examples are presented. But it is also noted that optimisation, integration and commercialisation of drug delivery systems based upon the biohybrid materials is still to be accomplished.

The next chapter of the second part is on "Biomimetic Systems" that are not really defined but illustrated by numerous examples. The final chapter of the second part is entitled "Nanostructured Scaffolds for Tissue Engineering". The discussion is focused on scaffolds on a nasnoscale level with a primary focus on the polymeric scaffolds. Extracellular matrix is overviewed first, followed by different fabrication and modification techniques used in nanoscaffolding. Applications examples are also given.

### Nanostructured therapeutic materials

It includes chapters on the "Hydrogel Nanocomposites for Intelligent Therapeutics", "Nanotechnology for Treating Bone Disorders" and "Nanotechnology and Cancer Therapy". The hydrogel nanocomposites are classified in accordance with incorporated nanomaterials. The mechanical and responsive properties are discussed as well as potential therapeutic applications. In the chapter on the bone disorder a good background to the topic is given. Two major applications of nanotechnology in bone disorder, nanopatterning of the implant surfaces and drug delivery to the bone tissue, are discussed. The final chapter of this part overviews the background knowledge on cancer, describes the oral delivery of anti-cancer drugs and focuses on hydrogels as nanomaterials that can be used in fighting cancer.

### Nanoparticulate systems in intelligent therapy

The final part contains five more chapters, mostly dealing with various forms of hydrogel materials. The first chapter is dedicated to dendrimers and star polymers, used for drug delivery, gene delivery, and chromophore encapsulation. These are very prospective materials but some open question exists like the biocompatibility of higher generation dendrimers and their behaviour in the body. Also, another interesting chapter is describing the shell cross-linked nanoparticles and their application in drug delivery.

### Verdict

On the whole it is a helpful collection of articles by leading specialists on some aspects of nanotechnology applications in medicine. It would be a useful reference book for a specialist interested to learn about hydrogels and other polymeric nanocarriers for drug delivery. It will be also a practical reference and study book for the students in pharmaceutical and medical fields, who are interested in the current state of affair of nanomaterials for therapeutics.

Some weak points are the assembly of mainly US authors and too much emphasis on hydrogels (the lead editor is a co-author in 7 out of 17 chapters). The division of the book in to parts and chapter titles are somehow a bit artificial, although it is clearly covering a lot of new interdisciplinary science, which makes it a difficult task to classify the material. The final chapter is obviously taken from the literature review in the doctoral thesis and some editing efforts should have been made here. But overall it is a useful and timely volume.
